# The role of co-parenting alliance as a mediator between trait anxiety, family system maladjustment, and parenting stress in a sample of non-clinical Italian parents

**DOI:** 10.3389/fpsyg.2015.01177

**Published:** 2015-08-19

**Authors:** Elisa Delvecchio, Andrea Sciandra, Livio Finos, Claudia Mazzeschi, Daniela Di Riso

**Affiliations:** ^1^Department of Developmental Psychology and Socialization, University of Padua, Padua, Italy; ^2^StarLab, Socio Territorial Analysis and Research, University of Padua, Padua, Italy; ^3^Department of Philosophy, Social and Human Sciences and Education, University of Perugia, Perugia, Italy

**Keywords:** co-parenting alliance, trait anxiety, parental stress, family maladjustment, structural equation modeling

## Abstract

This study investigated the role of co-parenting alliance in mediating the influence of parents’ trait anxiety on family system maladjustment and parenting stress. A sample of 1606 Italian parents (803 mothers and 803 fathers) of children aged one to 13 years completed measures of trait anxiety (State Trait Anxiety Inventory—Y), co-parenting alliance (Parenting Alliance Measure), family system maladjustment (Family Assessment Measure—III), and parenting stress (Parenting Stress Inventory—Short Form). These variables were investigated together comparing two structural equations model-fitting including both partners. A model for both mothers and fathers was empirically devised as a series of associations between parent trait anxiety (independent variable), family system maladjustment and parenting stress (dependent variables), mediated by co-parenting alliance, with the insertion of cross predictions between mothers and fathers and correlations between dependent variables for both parents. Results indicated that the relation between mothers and fathers’ trait anxiety, family system maladjustment and parenting stress was mediated by the level of co-parenting alliance. Understanding the role of couples’ co-parenting alliance could be useful during the family assessment and/or treatment, since it is an efficient and effective tool to improve the family system maladjustment and stress.

## Introduction

Parenting is a challenging process that involves complex variables not limited to caregiving activities (Bornstein, 2002). According to Belsky (1984) parenting behaviors are associated with three principal factors: child’s characteristics, family dimensions, and parent’s individual differences such as personality features and psychological resources. Personal differences would influence parenting competence more strongly than the other factors because they influence how people experience and respond to a wide variety of tasks (see, e.g., Caspi et al., 2005; Caspi and Shiner, 2006; Roberts et al., 2007). Furthermore, individual differences affect feelings and emotions toward parenting, and parent’s attributions to child behavior (Kochanska et al., 2004; Caspi et al., 2005; Belsky and Jaffee, 2006).

Co-parenting can be seen as a further indicator of parenting adjustment (Feinberg, 2003). Co-parenting (McHale, 1995; Feinberg, 2003) has been defined as a unique component of the marital relationship in which parents work together, or alternatively, struggle against each other when it comes to child rearing (McHale, 2007). Weissman and Cohen (1985) listed the following four characteristics for a good co-parenting alliance, which is one of the most important components of co-parenting: (1) both parents’ investment in the child, (2) evaluating reciprocal involvement with the child, (3) respect for each other’s judgment about child rearing, and (4) desire to communicate child-related information. Studies have shown how co-parenting alliance is positively associated with perceptions of parental support, marital relationship, as well as decreased stress, and, on the other hand, it has negative implications for parenting practices, and arguments about parenting practices (Abidin and Brunner, 1995; Sheras et al., 1998a,b; Stright and Bales, 2003; Schoppe-Sullivan et al., 2004; Askari et al., 2012; Kwan et al., 2015). A scarce level of co-parenting alliance influences family system adjustment and increases parenting stress (Morrill et al., 2010), defined as a feeling of poor parenting skills, a lack of freedom or restriction in certain aspects of the parent’s life, and a lack of social support (Abidin, 1995; Deater-Deckard and Scarr, 1996; Margolin et al., 2001). Several studies have demonstrated the mediating role of co-parenting in family functioning (Bonds and Gondoli, 2007; Feinberg et al., 2007a; Kwan et al., 2015), and how co-parenting has the potential to enhance family functioning and parent adjustment (Feinberg and Kan, 2008). Although some variables may serve either a moderating or mediating function, mediators are conceptually difference from moderators (Baron and Kenny, 1986). Whereas moderators are features that belong to individual prior to stressors, mediators become individual’s characteristics in response to stressors (Grant and McMahon, 2005).

Anxiety, besides being considered as a trait-stable indicator of parents’ personality (Majdandžić et al., 2012), is seen as an indicator of parenting and co-parenting adjustment. Anxiety might undermine parents’ ability to initiate and maintain positive affective interaction with other family members (i.e., the child, the partner); moreover, a disposition to experience anxiety might lead to intrusive and overprotective parenting. Studies have shown that anxious parents tend to report higher levels of parental distress and display higher levels of dysfunctional interactions (Dadds and Barrett, 1996; Hudson and Rapee, 2002). However, the extent to which specific parenting factors, and in particular trait anxiety, may affect family system have not been yet well assessed (Konold and Abidin, 2001; Majdandžić et al., 2012). Trait anxiety was also detected as an individual characteristic which impairs parenting alliance (Caldera et al., 2002). The links between parents’ characteristics, co-parenting relations, family maladjustment and parenting stress have been traditionally examined separately for fathers and mothers. Little is known about the relative contributions of these variables in the context of broader family models (Morrill et al., 2010). As an example, Kwan and colleagues, (2015) showed that parenting correlates impact differently in mothers and fathers. Although theorists argue the need to give space to both parents views, previous studies have emphasized the lack of data from fathers in family research (Bornstein, 2002; Bonds and Gondoli, 2007; Feinberg et al., 2007b; Kolak and Volling, 2007). For these reasons, in the current study, mothers as well as fathers’ contributions were taken into account.

Regarding possible clinical implication of the interplay between the dimensions discussed above, existing literature posited that articulation of adaptive family structure was determined by parents mental health and cohesiveness and it is strictly connected with the well-being of their children (Olson and Gorall, 2003). Disconnection and the lack of coordination between parents are some of the most important reasons for dysfunctional outcome in children since their first years of life (McHale et al., 2002).

**FIGURE 1:**
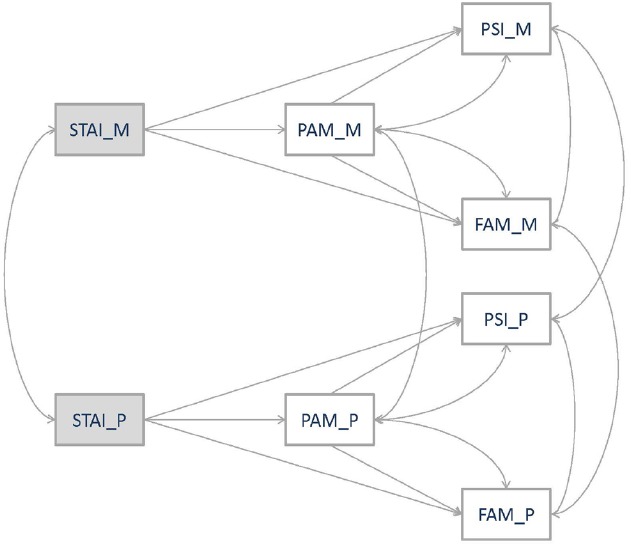
**Hypothetical model**.

The main purpose of our study was to empirically test the role of parental trait anxiety, mediated by co-parenting alliance on family system maladjustment and parenting stress, considering mothers and fathers simultaneously. To address this issue, structural equation modeling (SEM) was used to (a) test whether there exists a correlation between level of trait anxiety, co-parenting alliance, family maladjustment and parenting stress in fathers and mothers, (b) test whether mothers and fathers trait anxiety contributes to higher maladjustment and parenting stress as rated respectively by mothers and fathers, and (c) examine whether these hypothesized relationships were mediated by maternal and paternal co-parenting alliance. More specifically, the direct effect hypotheses supported that mothers and fathers’ trait anxiety and co-parenting alliance would predict greater family maladjustment and parenting stress as rated by mothers and fathers (Bonds and Gondoli, 2007). Measures of the same variables in fathers and mothers were expected to be related. An indirect relationship between trait anxiety and family maladjustment via co-parenting alliance was expected. A model is proposed to represent the hypothesized direct and indirect relationships of each parent’s trait anxiety and co-parenting alliance on parenting stress and family system maladjustment (Figure 1).

A parallel SEM was devised for both mothers and fathers and the contributions of both parents were simultaneously considered, being aware that empirical studies including members of the same parental couple are faced with the difficulty of studying data from non-independent members (Kenny et al., 2006).

## Materials and Methods

### Participants

The original sample included 956 parent couples. Statistical analyses, however, were carried out on the participants who filled the whole questionnaires. Self-reports of 153 participants showed one or more missing values, thus they were excluded. Missing data were especially due to slight parents’ inattention in filling the questionnaires. The final sample included 1,606 parents, 803 mothers and 803 fathers. They were married heterosexual couples of children from infancy to early adolescence (1–13 years old). Due to the large life-span included, parents were assessed considering their child developmental stage: (a) preschool children (1–5 years old) and (b) school aged children (6–13 years old).

Families were primarily recruited through day-cares, nurseries and schools, and met the following criteria: (a) both mothers and fathers agreed to participate, (b) all participants completed the entire assessment phase (c) parents and children did not meet criteria for psychiatric diagnosis and were not under psychological treatment. The mean age of mothers and fathers in this sample was 38.6 (SD = 5.74) and 40.92 (SD = 6.32) respectively. All subjects were Caucasian and lived in different regions of North and Central Italy. Parents’ socio-economic level, measured by SES (Hollingshead, 1975), was middle to upper for 91% of families, 7% had a medium to low socio-economic status and only 1% reported a very high level.

### Procedures

This study was conducted in compliance with the ethical standards for research outlined in the *Ethical Principles of Psychologists and Code of Conduct* (American Psychological Association, 2010). Approval by the Ethical Committee for Psychological Research was obtained from the University of Padova. Participation in the study was solicited via leaflets. Questionnaires were then distributed to 30 nursery school, 16 kindergartens, 12 elementary schools and four high schools in urban and suburban, located in North and Central Italy. Parents written signed informed consent to participate in the study were obtained before data collection. They completed the questionnaires at home and returned them to the research team through their children in a close envelope. Confidentiality was assured by replacing participant’s personal information with a numeric code. No incentives were awarded and voluntary participation was emphasized.

### Measures

*State-Trait Anxiety Inventory form Y* (STAI-Y; Spielberger et al., 1970). This measure is the gold standard for assessing anxiety in adults. It measures state and trait anxiety trough 40 items (20 each one) on a 4-point Likert scale. The scale showed good psychometric properties (Barnes et al., 2002). The Italian normative data comes from a large sample of 2304 subjects aged 16 to 60 years (Pedrabissi and Santinello, 1989). The subscale for trait anxiety evaluation (STAI-t) was used in this study.

*Parenting Alliance Measure* (PAM; Abidin, 1999; Abidin and Konold, 1999; Konold and Abidin, 2001) was used to measure co-parenting alliance. This 20-item self-report instrument assesses the strength of the perceived alliance of parents of children aged from 1 to 19 years. It assesses the parenting aspects of a couple’s relationship (e.g., how cooperative, communicative, and mutually respectful they are with regard to caring for their children). Parents responded to the items using a 5-points Likert scale ranging from 1 (strongly disagree) to 5 (strongly agree), with higher scores reflecting stronger co-parenting alliance and reciprocity in the parental role. PAM showed good psychometric characteristics and has been found to be stable for both mothers and fathers (Konold and Abidin, 2001; Delvecchio et al., 2014). The Italian validation was carried out by Delvecchio et al. (2014).

*Family Assessment Measure—III* (FAM-III General Scale; Skinner et al., 1983) is a 50-item self-report measure of family system maladjustment. It provides a multi-rater assessment of family functioning across universal clinical parameters. Participants are asked to answer on a 4-point Likert scale from 3 (strong agree) to 0 (completely disagree). High total scores revealed a maladaptive family functioning. The current study took into account only FAM-III Total score, which assesses family system shared values, norms and goals. The questionnaire showed good internal consistency for the total score (Van Riper, 2000). Discriminant validity studies reported an adequate sensitivity of the scale for detecting high-risk families (Jacob, 1991; Alderfer et al., 2008). Laghezza et al. (2014) carried out the Italian validation.

*Parenting Stress Index-Short Form* (PSI-SF; Abidin, 1995) is a 36-item measure designed to assess the overall level of stress experienced by parents. Core assumption of PSI-SF suggests that the level of stress in the parent–child dyad is the result of child, parent, and situational characteristics. The scores are based on a 5-point ordinal Likert scale from 1 (it does not fit for me) to 5 (it corresponds well for me). All 36 items are summed to yield a total score for parenting stress, a measure of parental state of helplessness. The measure was validated in several countries showing good psychometric characteristics (Reitman et al., 2002; Deater-Deckard, 2004; Haskett et al., 2006; McKelvey et al., 2009). Guarino et al. (2008) carried out the Italian validation.

### Data Analysis

The Statistical Package for Social Sciences (SPSS 21.0) was used to compute descriptive statistics, correlations, and to carry out analyses of variance (ANOVAs) on the overall score of trait anxiety (STAI-t), co-parental alliance (PAM), family system maladjustment (FAM-III) and parenting stress total scores (PSI-SF). SEM approach for observed variables was used to test the mediational effect of PAM on PSI-SF and FAM-III. LISREL 8 (Jöreskog and Sörbom, 1996) was used to estimate relations among the variables and assess model fit (Muthén and Muthén, 1998–2004). We also allowed for non-null correlations of errors among the same measures (i.e., mother and father) and within the same subject. Multiple criteria must be considered when evaluating model fit on the basis of various measures simultaneously, first, chi-square (χ^2^). A solution fits the data well when χ^2^ is not significant (*p* ≥ 0.05). This statistic, however, is sensitive to sample size; it can lead to rejection of a model differing very slightly from data for large samples, and, conversely, it can result in the acceptance of a model with salient differences from data for small samples. Therefore, Schermelleh-Engel et al.’s (2003) suggestions were followed which consider adequate a Chi-Square/df ratio lower than 3. The fit of the model was also assessed with the Comparative Fit Index (CFI), Non-Normed Fit Index (NNFI) and root mean square error of approximation (RMSEA; Kline, 2005). A CFI of 0.95 or above indicates a good fit, and below 0.90 indicates a poor fit. Also NNFI values greater than, or equal to, 0.95 indicate a good fit. If the RMSEA index is less than or equal to 0.05, the model is considered a good fit; values between 0.05 and 0.08 suggest reasonable error of approximation and if the index is greater than or equal to 0.10, the model is considered a poor fit. Finally, the choice of the best model was based on parsimony index, Akaike Information Criterion (AIC). The significance of the standardized path coefficients was determined by comparing the (absolute) t ratio to a critical t of 2.58 (*p* ≤ 0.01). Therefore the overall fit of the models was determined by using a combination of the results from the fit indexes, the significance of standardized path coefficients, and the significance of the indirect effect.

## Results

### Preliminary Analyses

Internal consistency for STAI-t, PAM, FAM-III and PSI-SF total scores were indexed by means of Cronbach’s alpha. Cronbach’s alpha for the STAI-T was adequate for Mothers α = 0.69 and for Fathers α = 0.70. Cronbach’s alpha for PAM were excellent (Mothers α = 0.93 and Fathers α = 0.92). FAM-III showed reasonable values (Mothers α = 0.75; Fathers α = 0.76). PSI-SF reported also high level of reliability (Mothers α = 0.93 and Fathers α = 0.94). Bivariate Pearsons’ correlations revealed that all scores were not significantly associated with the length of the spouses’ marriage, their income level, or either spouse’s education level. Therefore, these demographic variables were excluded from the analyses. As a first step, possible significant influences due to parental role (fathers versus mothers), child’s sex, and child age-group (preschool—1 to 5 years old-, versus school age—6 to 13 years old-) were assessed. Table 1 shows the means for STAI-t, PAM, FAM-III, and PSI-SF total scores in the whole sample, for fathers and mothers, and according to child gender and age group (preschool versus school children).

**TABLE 1:** **Means and Standard deviations for STAI-t, PAM, FAM-III, and PSI-SF according to parental role, child’s gender and age group (*N* = 1606)**.

****	**Parental Role**			**Gender**			**Age group**		
	**Mother (*n* = 803)**		**Father (*n* = 803)**		**Boys (*n* = 396)**		**Girls (*n* = 407)**		**(1–5 years) (*n* = 422)**		**(6–13 years) (*n* = 381)**	
	***M***	**SD**	***M***	**SD**	***M***	**SD**	***M***	**SD**	***M***	**SD**	***M***	**SD**
STAI-t	40.55	7.20	38.16	6.96	39.33	7.01	39.38	7.35	39.27	6.97	39.45	7.41
PAM	85.48	10.13	85.85	9.75	86.01	9.58	85.32	10.30	85.80	8.95	85.92	10.46
FAM-III	32.79	10.57	33.00	11.10	33.13	10.89	32.67	10.78	31.90	10.48	34.00	11.13
PSI-SF	69.94	18.03	67.03	17.43	67.49	17.56	68.48	17.95	68.04	15.85	67.91	19.66

STAY-t, trait anxiety; PAM, co-parenting alliance; FAM-III, family system maladjustment; PSI-SF, parenting stress.

Four analyses of variance (ANOVA) were performed on the total scores with parental role, children gender and age-group as between subject variables in order to verify if mothers and fathers showed similar levels of STAI-t, PAM, FAM-III and PSI-SF. According to Cohen’s suggestions (Cohen, 1992), partial eta-square estimates were considered to be substantially significant only within 1–5% effect sizes. Results of the ANOVAs are reported in Table 2. No significant differences were found according to children’s gender and age group for the considered variables. Focusing on parental role, the only significant result was found for STAI-t showing mothers reporting higher levels of anxiety than fathers, although their mean levels of trait anxiety were within the range of normative samples (Guarino et al., 2008). Furthermore, mothers reported higher levels of PSI-SF than fathers. However ${\text{η}}_{p}^{2}$ effect size of ANOVA was not within the 1–5% range, suggesting trivial results.

**TABLE 2:** **ANOVAs for STAI-t, PAM, FAM-III, and PSI-SF with parental role, child’s gender and age group as between subjects’ variables (*N* = 1606)**.

****	**Parental role**			**Child’s gender**			**Child’s age group**		
	*F*(1,1605)	***P***	${\text{η}}_{p}^{2}$	***F*_(1,1605)_**	***P***	${\text{η}}_{p}^{2}$	***F*_(1, 1605)_**	***P***	${\text{η}}_{p}^{2}$
STAI-t	30.01	0.00	0.02	0.23	0.63	0.00	0.27	0.61	0.00
PAM	1.84	0.18	0.00	1.59	0.21	0.00	0.25	0.62	0.00
FAM-III	0.00	0.96	0.00	0.49	0.48	0.00	15.44	0.00	0.01
PSI-SF	.15	0.01	0.00	1.49	0.22	0.00	0.03	0.86	0.00

STAY-t, trait anxiety; PAM, co-parenting alliance; FAM-III, family system maladjustment; PSI-SF, parenting stress.

The Pearson product-moment correlations between STAI-t, PAM, FAM-III, and PSI-SF were computed separately for mothers and fathers to study the associations among these variables. The correlations were all significant (*p* < 0.001). Correlation effect size was classified (Table 3) according to Cohen (1988): low effect size, if the Pearson’s *r* was lower than 0.30; medium effect size if *r* ranged between 0.31 and 0.50; and large effect size if *r* was higher than 0.50.

**TABLE 3:** ** Correlations of STAI-t, PAM, FAM-III, and PSI-SF total scores for mothers (*N* = 803) and fathers (*N* = 803)**.

****	**1**	**2**	**3**	**4**	**5**	**6**	**7**	**8**
1. STAI-t Mother	1							
2. STAI-t Father	20.32**	1						
3. PAM Mother	–0.34**	–0.27**	1					
4. PAM Father	–0.28**	–0.32**	.51**	1				
5. FAM-III Mother	0.45**	0.25**	–0.44**	–0.28**	1			
6. FAM-III Father	0.31**	0.40**	–0.37**	–0.42**	0.51**	1		
7. PSI-SF Mother	0.44**	0.19**	–0.30**	–0.23**	0.48**	0.23**	1	
8. PSI-SF Father	0.32**	0.47**	–0.35**	–0.45**	0.32**	0.46**	0.50**	1

STAY-t, trait anxiety; PAM, co-parenting alliance; FAM-III, family system maladjustment; PSI-SF, parenting stress. ** p < .01.

Medium effect size correlations were found for both parents between STAI-t and PSI-SF, suggesting that anxious parents tend to report higher levels of parental distress. As expected, PAM was negatively correlated with STAI-t, FAM-III, and PSI-SF.

### SEM with Observed Variables

**FIGURE 2:**
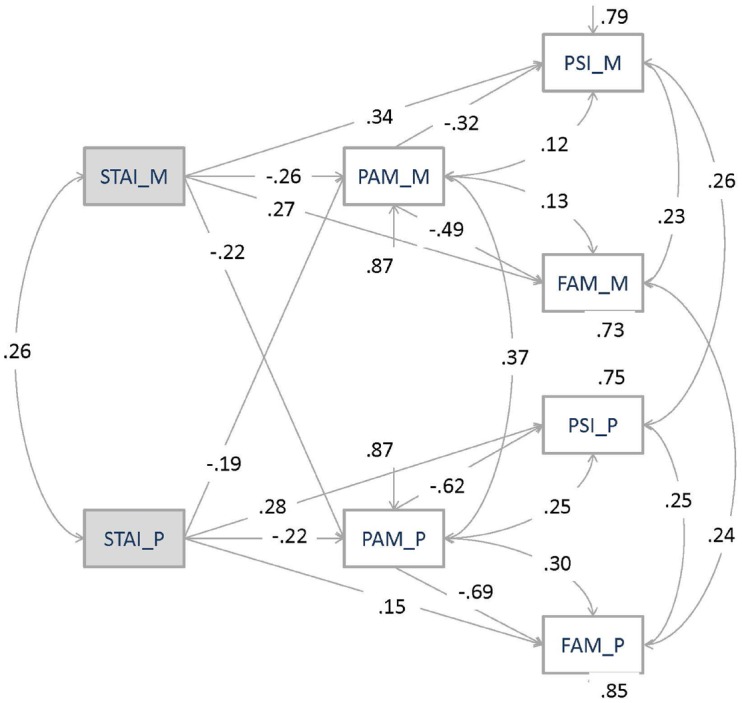
**Co-parenting alliance as a mediator of the effect of parental trait anxiety on family system maladjustment and parenting stress**.

In the present study two structural equation models were carried out. Both parents’ variables were inserted simultaneously within the two SEM tested. The first structural equation model (Model 1) was carried out to test the direct and indirect STAI-t effects as independent variable on FAM-III and PSI-SF path, with the insertion of PAM as a mediator factor. Correlations between dependent variables within the same parents were allowed. All standardized effects were significant. The CFI equal to 1.00 and the NNFI equal to 0.98 suggested a good fit. However, RMSEA equal to 0.123 and a ratio Chi-Square/df = 107.47/8 = 13.434 indicate a not adequate overall fit. Moreover, the presence of some not significant path coefficients underlined the need of a more adequate modified model with new paths of interactions between variables. Modification indices were taken into account in order to insert these new paths. These modifications led to Model 2. Model 2 was carried out starting from Model 1 structure with STAI-t as independent variable, PAM as mediator, FAM-III, and PSI-SF as predicted variables. However, in this model, mothers’ and fathers’ STAI-t was inserted as predictor of both mothers’ and fathers’ PAM. Direct and indirect predictions through PAM mediation of STAI-t were also considered. Not only mediational effects were considered for PAM but also its correlations with dependent variables of the same parent were taken into account. Correlations between FAM-III and PSI-SF were allowed within and between parents. The final model (Model 2) has been reached balancing among statistical requirements (e.g., modification indices) and interpretability of the resulting complex family system hypothesized and tested. Figure 2 showed standardized indirect and direct coefficients. Model 2 fits the data reasonably well as indicated by multiple indicators of fit: ratio Chi-Square/df = 18.16/6 = 3.026, RMSEA = 0.050, CFI = 1.00, and NNFI = 0.98. To evaluate the improvement of the fit from Model 1 and Model 2 AIC values were also compared (lower indicates a better fit, Schermelleh-Engel et al., 2003). The index strongly decreases from 160.65 to 78.21 for Model 2.

All the path coefficients demonstrated statistical significance (*p* ≤ 0.001). The results also showed that all the indirect effects between STAI-t, PAM, FAM-III, and PSI-SF were statistically significant both for mothers and fathers. Taken together, the results indicated that the relation between mothers’ trait anxiety, as well as fathers’ one, and family system maladjustment and parenting stress was mediated by co-parenting alliance level.

The model accounted for 13, 21, and 27% of the variance for mothers PAM, PSI-SF, and FAM-III, respectively. Among the fathers, the explained variance was 13, 25, and 15% for PAM, PSI-SF and FAM-III, respectively.

## Discussion and Conclusion

This study investigated the complex interplay between parental individual trait anxiety, mediated by co-parenting alliance on family system maladjustment and parenting stress, in a large sample of non-clinical Italian parents. Both parents were invited to take part in the study.

Results highlighted the good psychometric characteristics of the measures, showing adequate reliability for each selected tool. Moreover mothers and fathers appeared to be quite similar in terms of parental role, and according to their children’s age and gender.

Previous studies supported the idea that individual characteristics, such as trait anxiety, undermine family system, and that a scarce level of co-parenting alliance increase the risk of family maladjustment and parenting stress (Morrill et al., 2010). Starting from these theoretical-empirical bases, a structural equation model (Model 1) was hypothesized with the simultaneous insertion of both mothers and fathers variables. Because goodness of fit indices was not always satisfactory, a second model (Model 2) was carried out according to modification indices. In this model data fit was considered good and significantly higher than Model 1. Although, in an exploratory way, this model supported the ecologically complex interplay between trait anxiety, co-parenting alliance, family system maladjustment and parenting stress. Model 2 supported that trait anxiety—in mothers as well in fathers—was significantly predictive of the co-parenting alliance, for both partners. This result pointed out how each parent should account of the shared behaviors and practices of the couple that built the sense of co-parenting alliance.

Results of the current study have several important practical implications. Often family clinicians treat parent couples that are distressed in their co-parental relationship, which is often reinforced by powerful family dynamics. After assessing the family’s strengths and weaknesses, knowledge of this model could provide useful indications about which subsystem to target. For example, if the couple is primarily struggling with parenting stress, it may be effective to focus on their co-parental cohesion (in addition to parenting training), but it may also be effective to assess if parental stress was also undermined by parent personal anxiety. Furthermore, the viability of the model suggests that targeting couples’ co-parenting alliance could be an efficient and effective tool to influence family system maladjustment and stress. In other words, co-parenting interventions could have the power to contribute in diminishing their anxiety and stress. Prior research has demonstrated that co-parenting alliance is indeed a malleable construct, making such interventions feasible and practical (Cummings and Wittenberg, 2008; Feinberg and Kan, 2008). On the other hand, this amplified influence of co-parenting underscores the risks of leaving ineffective co-parenting unaddressed, because co-parenting dynamics have been shown to remain remarkably stable over time without intervention (McHale and Kuersten-Hogan, 2004). Given the systems focus on the field of family psychology, future family interventions, such as co-parenting treatments, may increasingly be developed to address multiple subsystems simultaneously.

Although the present study was carried out on a large sample of Italian parents, some limitations of this study must be considered in interpreting findings and proposing future lines of inquiry. The sample was quite homogeneous racially and socioeconomically, and reported being fairly satisfied in each of the family domains. For this reason, the generalizability of results is limited. It is important to investigate these effects in parents from more diverse and more highly distressed populations. Future studies should be carried out also with low-income, psychologically disadvantaged or high risk families in order to test the stability of the model tested, since characteristics like poverty, poor social milieu, psychological distress were found to affect the quality of parenting (Russel et al., 2008; Ciciolla et al., 2014).

The current design can only speak for the relationships between key variables, rather than comment on causal pathways. This conservative approach is appropriate given the exploratory nature of the project. The present study did not test the direction of causality among the variables of interest. These relationships should be examined in the context of a longitudinal study, which could provide stronger evidence of directionality or causality. Furthermore, it is of note that husbands and wives reported anxiety, co-parenting, and family system maladjustment and parenting stress quite differently, therefore, we were unable to constrain the parameter paths to equal each other in the models. Although a family-systems approach benefits from analytical methods such as those that incorporate both partners simultaneously, it is undoubtedly important to investigate gender differences as well. For instance, our finding that fathers’ family subsystems are more highly correlated, and accounted for more of the variance in their other subsystems than mothers’ ones, implies that gender differences are relevant in these processes. Further research may provide additional information about these gender differences. Additionally, this study used self-report measures only, making it difficult to separate true associations from common method variance. Data should be gathered using various methodologies in order to elucidate patterns accounting for the associations among these individual—and marital—level variables. This study examined only parenting variables. No attention was given to marital variables or to the “third part,” the child. Further explorations on the relationship between maternal and paternal measures involved in this study are necessary, mother and father measures of the parents’ involvement with the child, and measures such as child’s anxiety. The results may provide valuable contributions to the growing field of co-parenting research and the complex model empirically tested raises important practical implications for family system clinicians. This is one of the first studies according to our knowledge that investigates a path model of the interrelationships between anxiety, co-parenting alliance, family system maladjustment and parenting stress side-by-side. The model demonstrates the need for new conceptualizations of the co-parenting subsystem role to continue expanding our understanding of families. Researching the many roles of the co-parenting process for fathers and mothers has a theoretical and clinical importance that could contribute to this progress. Although preliminarily, this study empirically tested the variables simultaneously in a well-fitting model for mothers and fathers. The fitness of the model added empirical data, which supports the flexible and multiple roles that co-parenting can play in overall family systems. In conclusion, this exploratory study on Italian families provided new evidence to empirically support a developmental ecological model of mother’s and father’s views of themselves and their families.

Clinicians working with families need to recognize that parental interactions, which include the parents’ coparental capacities, reveal unique and important dimensions about the family’s functioning and health. Clinical evidence indicated that the presence of severe disengagement in the parental relationships has a great impact on psychosocial well-being of parents themselves and children. For these reasons, prevention and intervention programs tailored on children psychology health need to take into account also family assessment in terms of family functioning and alliance. Existing literature suggest that those data show an incremental value in understanding child maladaptive behaviors.

### Conflict of Interest Statement

The authors declare that the research was conducted in the absence of any commercial or financial relationships that could be construed as a potential conflict of interest.
